# Addressing Pain in Individuals with Neurocognitive Disorders Matters: A Literature Review

**DOI:** 10.1002/alz70858_107190

**Published:** 2025-12-26

**Authors:** Vanessa Jeune

**Affiliations:** ^1^ West Palm Beach Healthcare System, West Palm Beach, FL, USA

## Abstract

**Objective:**

In individuals with neurocognitive disorders (NCDs), pain management remains a significant concern, often left unaddressed. Progressive, irreversible cognitive decline combined with loss of independence can influence providers to overlook the necessity for improved pain assessment and management strategies in that vulnerable population. This review also highlights evidence‐based pharmacological and non‐pharmacological approaches to pain management to enhance health outcomes for persons with NCDs.

**Methods:**

A comprehensive literature review explored the correlation between pain, functional impairment, and cognitive decline. The author completed the following: examine common pain etiologies and their clinical manifestations in older adults with NCDs, analyze the efficacy of existing pain assessment tools and challenges healthcare professionals face in identifying pain, and evaluate the evidence‐based pharmacological and non‐pharmacological interventions for pain management in this population.

**Results:**

Pain in older adults with NCDs is often linked to comorbidities such as musculoskeletal conditions, neuropathies, and chronic illnesses exacerbated by lack of mobility. As pain persists and is left unattended, it can aggravate cognitive deficits and behavioral disturbances such as agitation, restlessness, and withdrawal. Traditional self‐report pain scales are ineffective, necessitating validated observational tools like the Pain Assessment in Advanced Dementia (PAINAD) scale and the Pain Assessment Checklist for Seniors with Limited Ability to Communicate (PACSLAC). Multimodal pain management approaches, such as the cautious use of non‐opioid analgesics, opioids when necessary, and adjuvant medications, have demonstrated efficacy. Non‐pharmacological strategies such as physical therapy, massage, music therapy, and cognitive‐behavioral interventions play a crucial role in pain relief.

**Conclusion:**

An elaborate but patient‐centered approach integrating specialized assessment tools and individualized treatment strategies is imperative to address pain, minimize behavioral disturbances, and improve the quality of life of individuals with NCDs. Healthcare providers must be educated on the tools to accurately assess and manage pain in this vulnerable population. Future research should focus on refining assessment tools and developing tailored interventions to meet individuals with cognitive impairments' unique needs.

